# Genotoxicity and Trace Elements Contents Analysis in Nile Tilapia (*Oreochromis niloticus*) Indicated the Levels of Aquatic Contamination at Three Egyptian Areas

**DOI:** 10.3389/fvets.2022.818866

**Published:** 2022-04-11

**Authors:** Ahmed H. El-Sappah, Mohamed M. Seif, Heba H. Abdel-Kader, Salma A. Soaud, Mohamed A. Abd Elhamid, Ahmed M. Abdelghaffar, Hamza H. El-Sappah, Huda Sarwar, Vivek Yadav, Pulak Maitra, Xianming Zhao, Kuan Yan, Jia Li, Manzar Abbas

**Affiliations:** ^1^School of Agriculture, Forestry and Food Engineering, Yibin University, Yibin, China; ^2^Genetics Department, Faculty of Agriculture, Zagazig University, Zagazig, Egypt; ^3^Toxicology and Food Contaminants Department, Food Industries and Nutrition Research Institute, National Research Centre, Giza, Egypt; ^4^National Institute of Oceanography and Fisheries (NIOF), Alexandria, Egypt; ^5^Faculty of Agriculture, Al-Azhar University, Assiut, Egypt; ^6^Department of Bioscience, University of Wah, Wah Cantt, Pakistan; ^7^College of Horticulture, Northwest A&F University, Xianyang, China; ^8^Institute of Dendrology, Polish Academy of Sciences, Kórnik, Poland

**Keywords:** trace elements, genotoxicity, Cd, Pb, qPCR, SDS-PAGE, Nile tilapia

## Abstract

The toxic waste and pollutants of heavy metals continuously pollute freshwater aquatic reservoirs, which have severe implications on aquatic life and human health. The present work aims to evaluate trace elements (Zn, Mn, Cu, Cd, and Pb) along with three sites, Mariout Lake, Abbassa, and River Nile Aswan in Egypt, using Nile tilapia (*Oreochromis niloticus*) as bioindicator. The quality assurance, health-risk assessment, sodium dodecyl sulfate-polyacrylamide gel electrophoresis (SDS-PAGE), quantitative polymerase chain reaction (qPCR), and micronucleus test were performed to investigate the effect of different trace elements on *Hsp70* gene level and micronuclei formation. We observed the highest expression of Hsp70 protein band of 70 KD and stress-responsive *Hsp70* gene in the liver followed by gills of Nile tilapia caught from Mariout and Abbassa, but the lowest expression was in Nile tilapia caught from Aswan. Obvious micronuclei were observed under the microscope in erythrocytes, and their number was gradually decreased in the following manner: Mariout > Abbassa > Aswan. Noticeably, Cu, Zn, and Mn contents were low. Still, Pb and Cd contents were higher than the toxicity level recommended by the Food and Agriculture Organization (FAO), The World Health Organization (WHO), and the European Commission (EC). These results showed that Hsp70's appearance at the two levels of mRNA and protein is an effective indicator for aquatic pollution besides the aberration at the chromosome level represented in the micronucleus test. Furthermore, these results showed that Nile tilapia of the Aswan region had comparatively low trace elements contamination and were suitable for consumption.

## Introduction

Untreated domestic wastewater, mining wastewater, industrial effluent discharge, metallurgical waste, excessive fertilizers, and pesticides in agriculture tremendously pollute the aquatic ecosystem and significantly affect human health (1). The Nile River is the main freshwater body that fulfills 97% of the irrigation and drinking necessities of Egyptian people (2). Additionally, the Nile River is a major source of fish hunting to fulfill the protein requirement of Egyptian people (3). On the other hand, industrial wastes comprising heavy metals are polluting freshwater aquatic resources in underdeveloped countries very seriously, which is subsequently affecting aquatic life, such as over-accumulation of toxic ingredients in animals being consumed as seafood (4). For example, discharge of untreated wastewater in the Nile River is contaminating and substantially changing the composition of freshwater, which became unfit for the growth of aquatic life and a serious cause of extinction of some economically important animal species (5).

Trace elements such as zinc (Zn), manganese (Mn), copper (Cu), and cadmium (Cd) are essential for enzyme activities. Still, excess of some trace elements such as Cd and Mn are persistent contaminants in the environment which are a serious cause of cancer and other fatal ailments in both animals and humans (6). Regionally, the primary source of Nile River aquatic pollution in Egypt is toxic industrial runoffs (7, 8). The heavy metal residues are retained for a long time in the soil and drained into aquatic reservoirs, absorbed by plants and aquatic animals, and cause a serious health threat to humans on ingestion (9, 10). These toxic trace elements are genotoxic pollutants (3, 11) and cause hepatorenal toxicity in humans on over-consumption (12, 13). It is a need of the hour to investigate the level of trace elements and evaluate genotoxicity level in animals such as Nile tilapia consumed as daily food, which is an inhabitant of the Nile River (3).

Different tilapia species are being used as a model to study environmental pollutants, such as heavy metals accumulation in an aquatic body (14–16). Fishes are the richest source of protein; moreover, fishes readily undergo heavy metals accumulation in their bodies (17, 18). Therefore, measuring the concentration of heavy metals accumulated in fish's bodies in an aquatic environment is an accurate indicator of heavy metal pollution of that aquatic ecosystem (19). Noticeably, Nile tilapia *Oreochromis niloticus* is dominant among all aquatic animals inhabiting the Nile River. Besides, it is one of the best and relatively inexpensive sources of protein because it is rich in essential amino acids, especially cysteine, as compared with other non-essential dietary proteins (3, 11). In light of guidelines published by the American Heart Association (AHA), fish should be added in daily life to prevent cardiovascular disease (20) because it is low in harmful fats, low-density lipids (LDL), and cholesterol and high in protein contents as compared with mutton, beef, chicken, and other sources (16).

The fish bioassay helps measure heavy metals contamination *via* the metal bio-magnification process (21). In freshwater, there are many physiological and oxidative stress biomarkers that depend on antioxidant enzymes, i.e., superoxide dismutase (SOD), catalase (CAT), and glutathione reductase (GR), as well as non-enzymatic antioxidants such as reduced glutathione (GSH) (22–24). The micronucleus (MN) frequency test is a robust technique among all predominant biomarkers to determine the cytogenetic damage caused by environmental toxicity and estimate water quality (3, 25). The MN test is employed to measure cytogenetic damage (26), eugenic and clastogenic effects, and genotoxicity ascribed to most of the toxic compounds (27). Thus, it is widely applied to fish species (28, 29). Real-time PCR is another highly sensitive, reproducible, and cost-effective technique used to measure the variable expression of genetic markers to identify DNA mutations (3, 30). Finally, sodium dodecyl sulfate-polyacrylamide gel electrophoresis (SDS-PAGE) is a widely used technique for protein separation on the basis of mass and charge ratio (m/e), which is also being employed in evaluation of genotoxicity (3, 31). In this study, some selected tissues of Nile tilapia were tested for contamination of inorganic heavy metals traces [lead (Pb), Cu, Cd, Zn, and Mn], their effect on genotoxicity, and expression pattern of *Hsp70* gene family. Finally, these results showed that Nile tilapia of the Aswan region had comparatively low trace elements contamination and is suitable for consumption. Furthermore, the effective use of *Hsp70* expression at the two levels (gene and protein) are rapid and cheap tools for detecting aquatic pollution.

## Materials and Methods

### Selection of Study Areas

**Figure 1:**
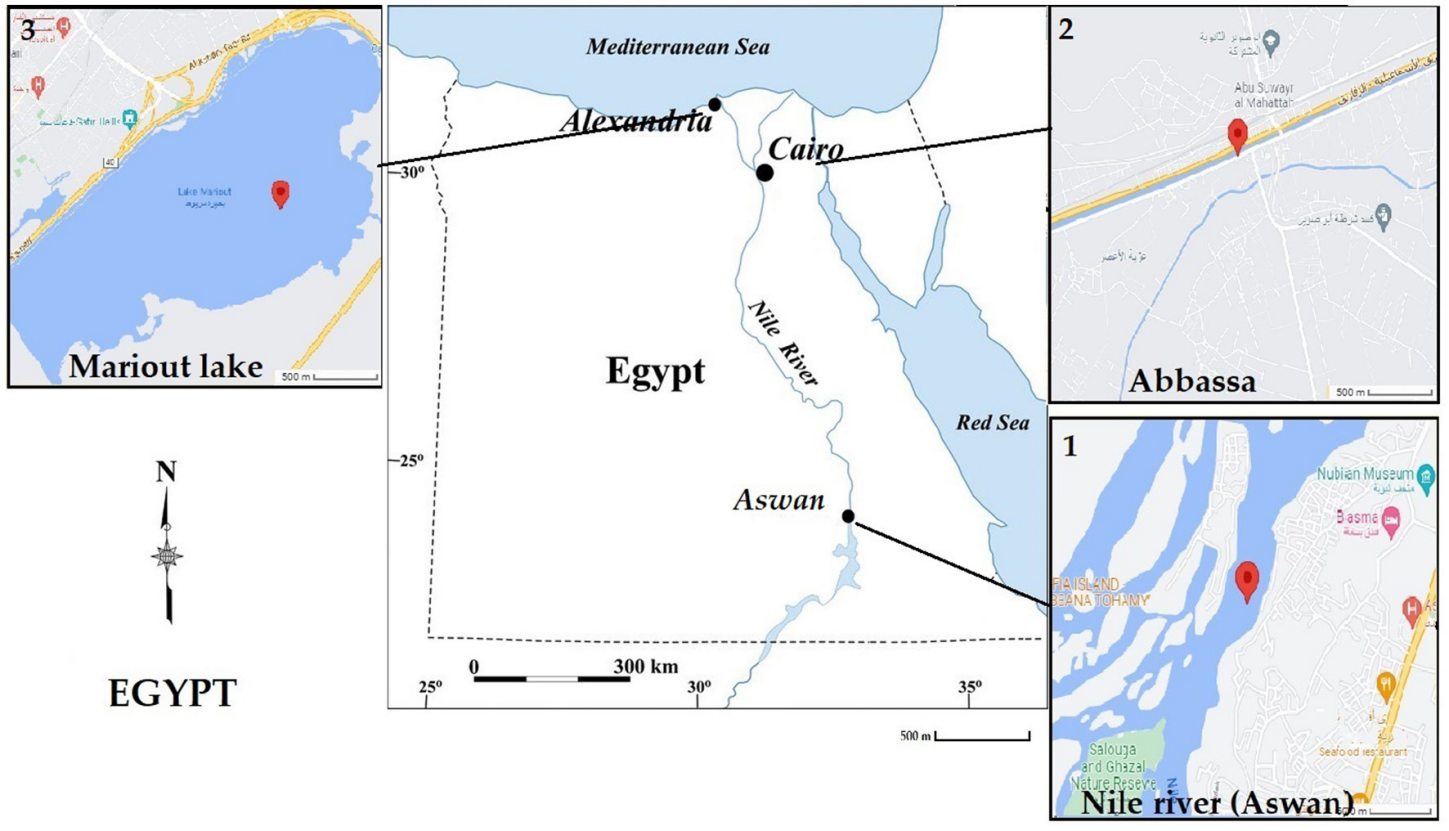
Egyptian map showing the three tested water sites, which are (1) Aswan, (2) Abbassa, and (3) Mariout.

In order to study the level and localization of trace elements in aquatic life, Nile tilapia were collected from three different geographical locations in Egypt, namely, Mariout Lake, Ismailia Canal (Abbassa), and Nile River (Aswan) (Figure 1). Mariout Lake is situated in the northern Nile Delta along southern Alexandria's Mediterranean coast, which covers ~200 km^2^ and is located at 31°09′17.7″N and 29°54′26.0″E. The Ismailia Canal (Abbassa) extends the Nile River, which flows from Shubra north of Cairo to the Suez Canal, passing by Ismailia city. We collected samples from the Abo-Swir region at 30°33′35.4″N and 32°06′51.9″E. Aswan city is situated at the bank of the Nile River with the following location: 24°04′33.1″N and 32°52′51.6″E. All these selected regions have different temperatures, diverse environmental conditions, and prime fishing zones for the whole country. Specifically, these regions are surrounded by industrial zones, which are the primary source of pollution.

### Samples Collection

A total of 90 freshwater Nile tilapia (30 fishes from each site), with length 10 ± 0.49 cm and weight 75 ± 0.5 g, were collected with the help of fishermen at the end of April 2018. Large tanks filled with aerated lake water were used to transport the fish to the physiology laboratory. After cessation of opercular movement, euthanasia was performed by dipping the fish for 10 min in a highly concentrated 10X MS222 solution of 250 mg/L during anesthesia (32, 33). The fish were thoroughly washed with distilled water and dissected on a clean glass surface to collect the following tissues: muscles, gills, and liver. Dissected tissues were packed in sterilized polythene bags, labeled, and stored at −20°C for further analysis.

### Determination of Trace Elements Contents

Nile tilapia tissues (1 g each) were dissected with a bladeless knife and dipped in a 100-ml tube containing 10 ml of concentrated HNO_3_:HCLO_4_ of 4:1 (16). For partial digestion, dry ashing of 0.5 g of each sample was performed on a hot plate at 60°C in a porcelain container by adding 5 ml of 2 N HCL. Subsequently, the mixture was placed on a hot plate to evaporate the liquid contents and then placed in a muffle furnace at 400–450°C overnight. The digested samples were dissolved in 0.5 ml HNO_3_ (10% solution, v/v), filtered, and immediately transferred to a 14-ml volumetric polypropylene tube; volume was completed with deionized water and placed at room temperature until examination (34). Similarly, blanks or negative controls were also prepared by following the same method without adding fish organ samples, and certified fish muscles (DOLT-4 dogfish liver) were used as a positive control. The samples were analyzed to determine Pb, Cd, Cu, Mn, and Zn by atomic absorption spectrophotometer (Perkin Elmer 3100). The heavy metal contents of each specimen were presented in μg/g wet weight. We analyzed data according to World Health Organization (WHO)/Food and Agriculture Organization (FAO) Legal Notice no. 66/2003 (http://faolex.fao.org/docs/pdf/eri42405.pdf) and European Commission (EC) (35). Commission Regulation as regards trace elements, Directive, 2001/22/EC, Npo: 466 regulations to record concentrations of trace elements present in wet weight of samples.

### Assurance of Quality

All acids and chemicals used in the whole experiment were of analytical grade, purchased from F. Maia Industry and Trade Ltd. (Sao-Paulo, Brazil) and Merck (Darmstadt, Germany). In order to decontaminate the materials, deionized water was prepared using a Milli-Q deionization system (Millipore, Bedford, MA, USA). All glassware were immersed for 24 h in a commercially available Extran®detergent (Merck, Darmstadt, Germany), then flushed with 10% nitric acid and subsequently with distilled water, decontaminated by washing with 0.5% KMNO_4_ solution (w/v), and finally washed with distilled water. For standard calibration, multi-element solutions comprising Zn, Mn, Cu, and Pb (10,000 mg L^−1^) were purchased from Ultra Scientific (Rhode Island, USA) and Cd (1,000 mg L^−1^) from SpecSol (Sao-Paulo, Brazil). The sample analysis results for quality control displayed that heavy metal determination level was satisfactory with 95–101% of certified recovery values of metals under study.

### Health Risk Assessment

To assess health risks, the estimated daily intake (EDI) of the trace elements Pb, Cd, Mn, Cu, and Zn *via* Nile tilapia fish consumption rate was compared with the current provisional tolerable daily intakes (PTDI). PTDI was calculated from the current provisional tolerable weekly intake (PTWI) by following method μg/kg body weight/day for 7 days (36). The average quantity of fish consumed per Egyptian person is 22.72 kg/year (16). The estimation of daily intake (EDI) of heavy metal elements per person can be performed by multiplying the average quantity of fish consumed per day per person by the mean concentration of measured heavy metal elements in fish.

Weekly intake of inorganic elements = concentration of an element in fish (μg/g wet weight) × mean fish consumption (g/person/week).

Weekly ingestion per body weight (kg) = weekly intake EDI per body weight (kg) reference consumer's body weight. Herein, a child as reference consumer is 15 kg, a young person as reference consumer is 40 kg, and an adult as reference consumer is 70 kg (16). According to the General Authority for Fish Resources Development (GAFRD), Cairo, Egypt, the overall consumption of fish per capita in Egypt was 351 g/week (37).

### Micronucleus Test

In order to perform a MN test to detect nuclear anomalies, peripheral blood was collected with a heparinized capillary tube from gills of Nile tilapia by following the already published Hooftman and De Raat (38) method. Blood droplets were smeared and fixed on glass slides by adding methanol. Feulgen staining was performed in the following method: The smears were first hydrolyzed in chilled 1 N HCl for 15 min at 60°C, rinsed with cold 1 N HCl for a few seconds, and washed with ddH_2_O for a few seconds to remove extra HCl. Erythrocyte staining was performed by adding Schiff's solution and placing the smears at room temperature for 1 h, then washing the smears twice with newly prepared bisulfite solution (10% K_2_S_2_O_5_, 5 ml HCl, and 100 ml ddH_2_O). Finally, the smears were counterstained by adding 1% aqueous light green solution for 1 min, washed with ddH_2_O for a few seconds, and mounted in the digital picture exchange (DPX).

Approximately 5,000 erythrocytes were examined from each fish and six fishes for each trace element to detect and score the micronuclei in erythrocytes at ×1,000 magnification using an oil immersion lens (39). In order to avoid stain particles being scored, only non-retractable particles were scored as abnormalities. The cells with intact nuclear and cell membranes which have similar central nucleus staining with their size less than one-third of the main nucleus were scored. Moreover, MN exists marginally, and overlapping the main nucleus was challenging to identify their nuclear boundaries. Noticeably, MN staining should be similar to staining of the main nucleus. MN frequency was also calculated according to the following equation:

MN % = (Number of cells with micronuclei)/(Total number of cells) ×100

### Protein Extraction and SDS-PAGE

In order to extract total protein contents, ~0.1 g fresh muscle tissues of three Nile tilapia from each fishing zone were used. Samples were ground to disrupt the cell membrane, suspended in 1.0 ml of lysing buffer (Proteinase K, ThermoFisher, USA), subsequently heated at 100°C for 5 min, and centrifuged at 10,000 rpm for 30 min to sediment coarse particles. To separate proteins on the basis of charge and mass (e/m) ratio, 2 μl of each sample was loaded in SDS-PAGE gel along with a protein marker (3). The Gel-Pro Analyzer package V3.1 analyzed the protein molecular mass (Media Cybernetica 1993–97).

### The Expression Level of the *Hsp70* Gene

In order to investigate the change in the expression level of the *Hsp70* gene, total RNA and protein contents were extracted from muscle tissues, gills, and liver of Nile tilapia by using Trizol reagent (Invitrogen, USA). The concentration of total RNA was quantified on a Nanodrop spectrophotometer (ThermoFisher Scientific Inc., Wilmington, DE, USA), and quality was determined by running 1% agarose gel (40). Total extracted RNA was treated with RNase-free DNaseI to remove DNA. Then 2 μg of total RNA was used for cDNA synthesis by using First Strand cDNA Kit (Invitrogen, Waltham, MA, USA) following the manufacturer's protocol. β*-actin* and *Hsp70* primers were designed using Primer Premier 5.0 software in quantitative polymerase chain reaction (qPCR) for gene expression analysis (Table 1) (41). qPCR was performed following an already published protocol (42). qPCR profile was adjusted as follows: 95°C for 10 min, 95°C for 15 s, and 60°C for 60 s with 40 cycles. The threshold cycle (C_T_) method was used to analyze relative gene expression level, normalized with a geometric mean of expression of housekeeping gene β*-actin* (43), and finally calculated by 2^−Δ*ΔCt*^ equation (44, 45). Total protein was boiled at 100°C with Coomassie Brilliant Blue buffer and centrifuged at full speed for 5 s, and 2 μl of supernatant was loaded in SDS-PAGE gel prepared 1 day before along with a protein marker.

**Table 1:** The qPCR primers designed by Primer Premier 5.0.

**Target gene**	**Gene descriptions**	**Primer sequence 5–3**	**Product length (bp)**
HSP70	Heat shock protein 70	F: GCATTCACACCATGAGGCGTT	283
		R: GCTTTGACA CGCTTCCCATT	
Actin	β-Actin	F: CTACAATGAGCTGCGTGTGG	143
		R: AAGGAAGGCTGGAAGAGTGC	

### Ethical Statement

Catching of Nile tilapia fish was performed by following the standard ethical conduct guidelines for animals excluding human beings for research purposes published by the NIOF, Egypt, and the American Psychological Association for animal research and ethics in 2010–11.

### Statistical Analyses

Statistical Processor System Support (SPSS 20, Armonk USA) and Microsoft Excel (version 2013) were employed to analyze data statistically using one-way analysis of variance (ANOVA) and least significant difference (LSD) test at significance level *p* < 0.05. The data were recorded as mean ± standard deviation (mean ± SD), where *n* = 6.

## Results

### Trace Elements in Different Organs of Nile Tilapia

The concentrations of the trace elements Pb, Cd, Cu, Mn, and Zn were analyzed in the muscles, gills, and liver tissues of Nile tilapia fish caught from Aswan, Abbassa, and Mariout Lake in Egypt. The order of average concentrations of all five trace elements recorded in Nile tilapia was Zn > Mn > Cu > Cd > Pb and significantly higher in the liver tissues of all fishes caught from the three different locations. The concentrations of trace elements in different tissues of Nile tilapia from Mariout Lake were in the following orders: in the liver, Zn > Cu > Mn > Pb > Cd; in gills, Zn > Mn > Cu > Cd > Pb; and in muscles, Zn > Pb > Mn > Cd > Cu. Similarly, the orders of concentrations of all five trace elements in different tissues of Nile tilapia from Abbassa were as follows: in the liver, Zn > Cu > Cd > Mn > Pb; in gills, Zn > Mn > Pb > Cu > Cd; and in muscles, Zn > Mn > Cd > Pb > Cu. Finally, the orders of concentrations of all five trace elements in different tissues of Nile tilapia from Aswan were as follows: in the liver, Zn > Cu > Mn > Pb > Cd; in gills, Zn > Mn > Cu > Pb > Cd; and in muscles, Zn > Pb > Mn > Cu > Cd (Table 2).

**Table 2:** Concentrations of trace elements in muscles, gills, and liver (μg/g wet weight) of Nile tilapia from three different sites in Egypt.

**Trace element residues (μg/g)**	**Location**	**Pb**	**Cd**	**Cu**	**Mn**	**Zn**
Muscles (μg/g)	Aswan	0.34 ± 0.08	0.09 ± 0.9	0.23 ± 0.05	0.05 ± 0.04	1.31 ± 0.52
	Abbassa	0.53 ± 0.10^a^	0.7 ± 0.46^a^	0.37 ± 0.04	0.99 ± 0.05	2.80 ± 0.30
	Mariout	2.7 ± 0.25^b^	1.17 ± 0.15^b^	0.47 ± 0.05	1.41 ± 0.18	3.9 ± 0.17
Gills (μg/g)	Aswan	1.87 ± 0.16^a^	0.40 ± 0.18	2.01 ± 0.82	3.81 ± 1.85	16.41 ± 1.37
	Abbassa	3.19 ± 0.67^b^	2.79 ± 0.93^b^	2.91 ± 0.93	20.45 ± 2.0	22.8 ± 2.39
	Mariout	5.39 ± 0.22^a^	7.81 ± 0.33^b^	8.11 ± 0.45^a^	23.4 ± 0.19	28.71 ± 4.1
Liver (μg/g)	Aswan	0.78 ± 0.56^a^	0.61 ± 0.45^a^	2.76 ± 1.03	0.81 ± 1.08	21.5 ± 1.1
	Abbassa	1.46 ± 0.37^a^	1.76 ± 0.96^b^	13.81 ± 1.22^a^	3.02 ± 0.29	50.13 ± 5.4^a^
	Mariout	3.48 ± 0.22^b^	2.98 ± 0.33^b^	19.6 ± 0.45^b^	3.62 ± 0.19	59.49 ± 4.1^a^
International standard of permissible limits	FAO, 1983	0.5	0.5	30	-	30
	FAO/WHO, 1989	0.5	0.5	30	-	40
	WHO, 1989	2	1	30	1	100
	EC, 2006–2011	0.3	0.05	-	-	50

*Data are expressed as mean ± SD (n = 6), and mean values with different letters for each metal in the same row are significantly different (LSD post-hoc test, p < 0.05)*.

The overall concentrations of all five trace elements Zn, Cu, Mn, Pb, and Cd in Nile tilapia tissues collected from all three locations in Egypt were in the order liver > gills > muscles. The Pb content in different tissues of Nile tilapia from all three locations was in the order gills > liver > muscle, Cu and Zn contents were in the order liver > gills > muscles, Mn content was in the order gills > liver > muscles, and Cd content in different tissues of Nile tilapia from Aswan and Abbassa was in the order liver > gills > muscle and in Nile tilapia from Mariout was in the order gills > liver > muscles. The concentration of trace elements in muscle tissues was usually more minor than in the liver and gills. The overall accumulative concentration of trace elements in Nile tilapia organs was in the order Mariout Lake > Abbassa > Aswan. The highest concentration of trace element was of Zn in the liver tissues of Nile tilapia from Mariout Lake, while the lowest concentration of trace element was of Mn, which was recorded in muscle tissues of Nile tilapia from Aswan (Table 2).

### Lead

The Pb content in muscle tissues of Nile tilapia caught from Aswan was 0.34 ± 0.08 μg/g, from Abbassa was 0.53 ± 0.10 μg/g, and from Mariout was 2.7 ± 0.25 μg/g. The Pb content in gills of Nile tilapia caught from Aswan was 1.87 ± 0.16 μg/g, from Abbassa was 3.19 ± 0.67 μg/g, and from Mariout was 5.39 ± 0.22 μg/g. Similarly, the Pb content in liver tissues of Nile tilapia caught from Aswan was 0.78 ± 0.56 μg/g, from Abbassa was 1.46 ± 0.37 μg/g, and from Mariout was 3.48 ± 0.22 μg/g. The average concentration of Pb in different tissues of Nile tilapia ranges between 0.34 ± 0.08 and 5.39 ± 0.22 μg/g. The highest concentration of Pb was recorded in gills of Nile tilapia caught from Mariout Lake and the lowest in muscle tissues of Nile tilapia caught from Aswan. The mean Pb content recorded in this study was higher than the permissible level (Table 2).

### Cadmium

The Cd content in muscle tissues of Nile tilapia caught from Aswan was 0.09 ± 0.9 μg/g, from Abbassa was 0.7 ± 0.46 μg/g, and from Mariout was 1.17 ± 0.15 μg/g. The Cd content in gills of Nile tilapia caught from Aswan was 0.40 ± 0.18 μg/g, from Abbassa was 2.79 ± 0.93 μg/g, and from Mariout was 7.81 ± 0.33 μg/g. Similarly, the Cd content in liver tissues of Nile tilapia caught from Aswan was 0.61 ± 0.45 μg/g, from Abbassa was 1.76 ± 0.96 μg/g, and from Mariout was 2.98 ± 0.33 μg/g. The mean concentrations of Cd in all three tissues of Nile tilapia were ranged between 0.09 ± 0.9 and 5.39 ± 0.22 μg/g. The highest concentration of Cd was recorded in gills of Nile tilapia caught from Mariout and the lowest in the muscle tissues caught from Aswan. In our study, the mean Cd concentration surpassed the permissible limit (Table 2).

### Copper

The Cu content in muscle tissues of Nile tilapia caught from Aswan, Abbassa, and Mariout was 0.23 ± 0.05, 0.37 ± 0.04, and 0.47 ± 0.05 μg/g, respectively. The Cu content in gill tissues of Nile tilapia caught from Aswan, Abbassa, and Mariout was 2.01 ± 0.82, 2.91 ± 0.93, and 8.11 ± 0.45 μg/g, respectively. Similarly, the Cu content in liver tissues of Nile tilapia caught from Aswan, Abbassa, and Mariout was 2.76 ± 1.03, 13.81 ± 1.22, and 19.6 ± 0.45 μg/g, respectively. The mean concentration of Cu in various tissues of Nile tilapia was varied between 0.23 ± 0.05 and 19.6 ± 0.45 μg/g. The mean concentration of Cu in this study did not exceed the international recommended standard limits (Table 2). The highest concentration of Cu was recorded in the liver of Nile tilapia caught from Mariout and the lowest in the muscle tissues caught from Aswan.

### Manganese

The Mn content in muscle tissues of Nile tilapia caught from Aswan, Abbassa, and Mariout was 0.05 ± 0.04, 0.99 ± 0.05, and 1.41 ± 0.18 μg/g, respectively. The Mn content in gills of Nile tilapia caught from Aswan, Abbassa, and Mariout was 3.81 ± 1.85, 20.45 ± 2.0, and 23.4 ± 0.19 μg/g, respectively. Similarly, the Mn content in liver tissues of Nile tilapia was 0.81 ± 1.08, 3.02 ± 0.29, and 3.62 ± 0.19 μg/g from Aswan, Abbassa, and Mariout, respectively. The mean concentration of Mn in all tissues of Nile tilapia was recorded between 0.05 ± 0.04 and 23.4 ± 0.19 μg/g. The highest concentration of Mn was recorded in gills of Nile tilapia caught from Mariout and lowest in the muscle tissues caught from Aswan. The Mn content recorded in Nile tilapia in our study did not exceed the recommended level (Table 2).

### Zinc

The Zn content in muscle tissues of Nile tilapia caught from Aswan, Abbassa, and Mariout was 1.31 ± 0.52, 2.80 ± 0.30, and 3.9 ± 0.17 μg/g, respectively. The Zn content in gills of Nile tilapia caught from Aswan, Abbassa, and Mariout was 16.41 ± 1.37, 22.8 ± 2.39, and 28.71 ± 4.1 μg/g, respectively. Similarly, the Zn content in liver tissues of Nile tilapia caught from Aswan was 21.5 ± 1.1 μg/g, from Abbassa was 50.13 ± 5.4 μg/g, and finally from Mariout was 59.49 ± 4.1 μg/g. The concentration of Zn recorded in different tissues of Nile tilapia ranged between 1.31 ± 0.52 and 59.49 ± 4.1 μg/g. The highest Zn content was recorded in the liver of Nile tilapia caught from Mariout and the lowest in the muscle tissues caught from Aswan. The mean Zn residues recorded in this study did not surpass the recommended level (Table 2).

### Health Risk Assessment

The EDI of the trace elements Pb, Cd, Cu, Mn, and Zn (μg/kg body weight/week) through consumption of Nile tilapia caught from the three sites Aswan, Abbassa, and Mariout by Egyptians was estimated and compared with PTDI (Table 3). This study revealed that the EDI of Pb, Cd, Cu, Mn, and Zn by ingesting Nile tilapia caught from Aswan, Abbassa, and Mariout were less than the PTDI recommended by FAO/WHO (Table 3). In contrast, high Pb residues in Nile tilapia caught from Mariout and high Cd residues in Nile tilapia caught from Abbassa and Mariout were consumed by children and young people, so their EDI was more significant than the PTDI (Table 3). We calculated the daily quantity of Nile tilapia to attain proper daily metal intake PTDI for a person of 15, 40, and 70 kg (Table 3).

**Table 3:** Estimated daily intakes (EDI) of trace elements (μg/day/person) in Nile tilapia consumed by a child, young person, and adult caught from three different sites in Egypt.

**Location**		**Pb**	**Cd**	**Cu**	**Mn**	**Zn**
EDI (μg/day/person 15 kg) (a child)	Aswan	1.411 (37.95)	0.37 (166.6)	0.95 (32,608)	0.2 (77,600)	5.43 (11,450.3)
	Abbassa	2.19 (24.45)	2.90^a^ (21.42)	1.53 (20,270)	4.1 (3,919.1)	11.62 (5,357.14)
	Mariout	11.20^a^ (4.78)	4.8^a^ (12.82)	1.95 (15,957.4)	5.85 (2,752.1)	16.18 (3,846.1)
EDI (μg/day/person 40 kg) (young)	Aswan	0.529 (420)	0.14 (444.4)	0.35 (86,956.5)	0.07 (228,560)	2.03 (30,534)
	Abbassa	0.824 (269.4)	1.08^a^ (57.1)	0.57 (54,054.05)	1.54 (11,543.43)	4.35 (14,285.7)
	Mariout	4.20^a^ (52.88)	1.82^a^ (34.18)	0.73 (42,553.19)	2.19 (8,104.9)	6.06 (10,256.4)
EDI (μg/day/person 70 kg) (adult)	Aswan	0.3 (735)	0.08 (777.77)	0.2 (152,173)	0.04 (399,980)	1.16 (53,435.1)
	Abbassa	0.471 (269.4)	0.62 (100)	0.329 (94,594)	0.88 (2,001)	2.49 (25,000)
	Mariout	2.40^a^ (92.55)	1.04 (59.82)	0.417 (744,680.8)	1.25 (14,183.6)	3.46 (17,948.7)
PTDI		3.57	1	50	285.7	1,000
PTDI_15_		53.55	15	7,500	3,880.5	15,000
PTDI_40_		142.8	40	20,000	11,428	40,000
PTDI_70_		249.9	70	35,000	19,999	70,000

*The values in brackets are healthy daily intake of Nile tilapia that should not be exceeded to maintain the PTDI of each element for 15, 40, and 70 kg person = PTDI × BW/metal concentration (μg/g) according to FAO/WHO (35). PTDI15 = PTDI × 15 kg, PTDI40 = PTDI × 40 kg, and PTDI70 = PTDI × 70 kg. Data are expressed as mean ± SD (n = 6) (LSD post-hoc test, p < 0.05)*.

*^a^These EDI values are in accordance with tolerable concentrations published by FAO/WHO (35)*.

### Micronucleus Test

**Figure 2:**
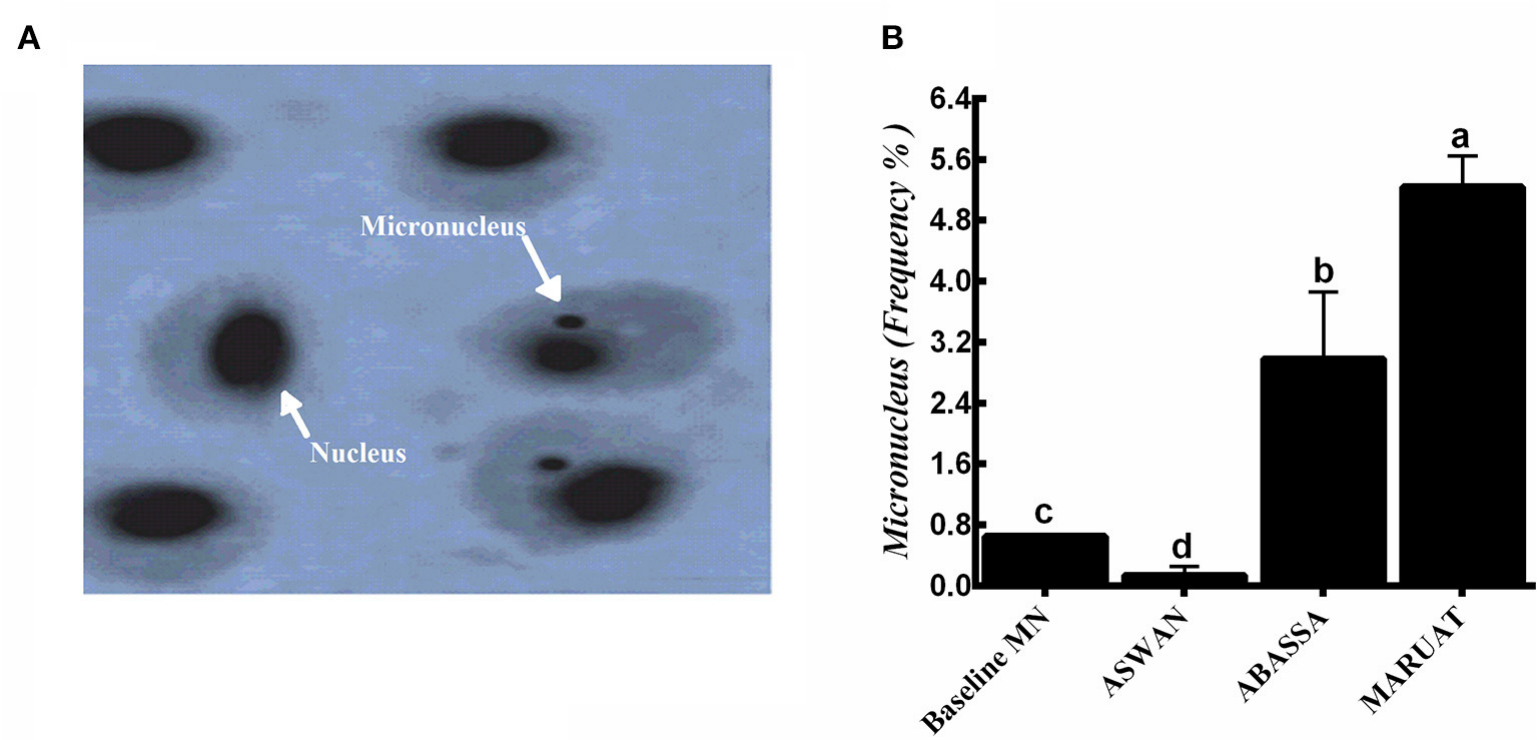
**(A)** Images taken by oil immersion lens of (1) normal erythrocyte, and samples from (2) Aswan, (3) Abbassa, and (4) Mariout of Nile tilapia fish (×1,000) = 10 μm and **(B)** micronucleus frequencies in the three tested sites compared with the baseline (0.65%). The different letters represent statistically significant differences (*P* < 0.05) between treatment and control.

Biosynthesis of MN in erythrocytes harvested from Nile tilapia from the three different locations on the Nile River, which were Aswan, Abbassa, and Mariout, was observed at ×1,000 magnification under a microscope using an oil immersion lens. Higher MN number represents the highest level of trace elements contamination and other trace pollutants in the body of aquatic animals. Apparent small nuclear anomalies, also known as micronuclei, were observed, which were present in adjacent form with the nucleus (Figure 2A). We observed that the order of concentrations of MN induction in fishes was Mariout > Abbassa > Aswan, with frequencies of 5.24, 2.89, and 0.14% compared with baseline 0.65% (Figure 2B).

### SDS-PAGE and qPCR Analysis of *Hsp70* in Different Tissues of Nile Tilapia

**Figure 3:**
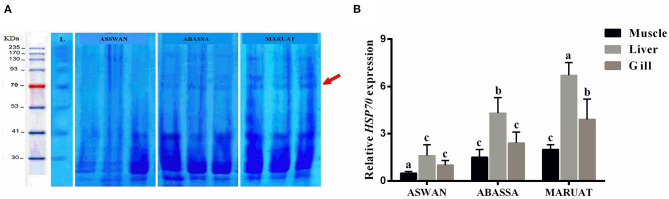
**(A)** SDS-PAGE of protein loci. Lane M: PageRuler™ Plus pre-stained protein ladder Fermentas and **(B)** relative HSP70 gene expression for Nile tilapia from three studied areas. The different letters represent statistically significant differences (*p* < 0.05) between treatment and control.

In order to analyze differential gene expression under trace elements stress, first of all, total protein content and then RNA was extracted from muscles, liver, and gills tissues of Nile tilapia caught from each experimental site in triplicate manner. Total protein from each sample was boiled at 100°C with Coomassie Brilliant Blue buffer and loaded in SDS-PAGE gel along with a protein marker. We observed very obvious Hsp70 protein bands of 70 KD in muscle, liver, and gill tissues of Nile tilapia caught from Mariout and Abbassa, but an Hsp70 protein band was absent in Nile tilapia muscle, liver, and gill tissues caught from Aswan (Figure 3A). Subsequently, the expression level of *Hsp70* genes was evaluated in three tissues of Nile tilapia—muscle, liver, and gills—by employing qPCR. The overall highest expression of *Hsp70* genes was observed in liver tissues in all samples, followed by gills and muscles (Figure 3B). According to sampling location, the highest expression of *Hsp70* genes in different tissues of Nile tilapia was in the manner liver > gill > muscles (Figure 3B).

## Discussion

Specific concentrations of trace elements such as Zn, Mn, and Cu are an essential part of animals and the human diet, which play a significant role in growth and development. In contrast, toxic elements such as Cd and Pb have negative effects. Analysis of bioaccumulation of these trace elements in the fish organs, which is a rich source of protein, is highly recommended for early detection of the concentration of toxic elements. Moreover, metals are easily dissolved in water then subsequently absorbed by fish and invertebrates, inducing a wide range of biological effects, from essential to lethal for living organisms. After heavy metal is ingested, it is then transported into different body compartments, i.e., organs and blood, by lipoproteins where they specifically can be directed to different centers: (a) *action centers* where an endogen macromolecule such as protein or a certain cellular structure interacts with the toxic metal; (b) *metabolism centers* where detoxified enzyme acts on the metal; (c) *storage centers* where the metals are collected in a toxic inactive state; and (d) *excretion centers* to dispose of the metals. The lethal effects of metals in aquatic animals were induced by inhibiting cellular respiration enzymes (46).

Tilapia fish was used as a model organism to investigate bioaccumulation of trace elements caught from Mariout Lake, Abbassa, and Aswan in Egypt and analyzed to disclose the level of genotoxicity caused by Zn, Mn, Cu, Cd, and Pb. Currently, Lake Mariout is considered a highly polluted wetland in north Egypt (47). The primary cause of the critical degradation is its proximity to Alexandria City and industrial and human activities (e.g., urban wastewater discharges and agricultural drainage). However, fishing remains one of the major activities on Lake Mariout, as it represents high fishing rates in the past (48). The higher concentrations of metals in sediments of Abbassa is due to the application of animal manures, which are high in concentrations of metals (49). At the same time, the River Nile in Aswan is lowly contaminated and polluted except for a few sites nearest to pollution resources (50). The estimation of trace metals intake is an indicator of health risk assessment. It can be compared with the permissible tolerable recommended intake level by international committees such as FAO and WHO. Our findings demonstrated that the estimated weekly intake (EWI) of Cd and Pb were safe for adults and youth but not suitable for children who consume Nile tilapia, similar to *Clarias gariepinus* (16).

Cu, Mn, and Zn are essential trace elements required for proper metabolic and enzyme activities. Cu is an essential trace element that serves as a co-factor for the biosynthesis of hemoglobin and other enzymatic activities (51). Still, excessive consumption of Cu (1.5–3 mg) can cause poisoning, blood vomit, sickness, gastrointestinal pain, diarrhea, liver damage, fever, and Wilson's disease (52). The highest concentration of Cu was 19.6 μg/g recorded in liver tissues of Nile tilapia, which was significantly higher as compared to 0.01 μg/g in *Cyclocheilichthys apogon* and 0.05 μg/g in *Hampala macrolepidota* caught from the mining pool, Selangor (53, 54). The Cu, Mn, and Zn contents were below permissible human consumption limits according to FAO/WHO (Table 2), and these findings agree with El-Moselhy (55).

Mn is essential for bone structure, reproduction, and regular activity of enzymatic reactions (56, 57). Skeletal and reproductive disorders are mainly caused by lack of Mn (58, 59). However, excessive consumption of Mn may lead to psychological and neurological disturbances. According to the UK Community of Expert on Vitamins and Minerals and the Food and Drug Administration (FDA), high intakes of Mn (up to 200 μg/kg) cause manganism (Parkinson's disease). The highest Mn content 23.4 μg/g was recorded in gills which was significantly higher than 5.61 μg/g in the liver of Nile tilapia caught from the Nile River, Egypt (8), but significantly lower than 735.84 μg/g in the gills of *Chrysichthys nigrodigitatus* (1). Zn is required for optimal animal growth, reproduction, and survival of animals and humans (60, 61). Higher consumption of Zn (50 mg/day) can cause poisoning, severe diarrhea, and fever (52, 62); interferes with the availability of Cu to the body by stimulating the synthesis of metallothionein in the digestive tract (63); and lowers immune response and high-density lipoprotein (HDL) level (64, 65). The highest Zn content, 59.49 μg/g, was recorded in liver tissues, significantly higher than 0.434 μg/g in the muscle tissues of Nile tilapia and 0.14 μg/g in *Nemurus* caught from a mining pool in Beranang, Selangor (53, 66). The leading causes of Zn toxicity are smelting, mining, and wastewater disposal (67). The EDI of Cu, Mn, and Zn trace elements for children, youth, and adults consuming Nile tilapia in Egypt were less than PTDI recommended by FAO/WHO.

Cd and Pb are not essential for growth and development and often have adverse effects on the nervous system and cause renal failure, liver injury, slowdown of reflex arc, demineralization of bones, lung cancer, stomach cancer, coma, and even death; i.e., Cd 2.4–10 μg/g of creatinine causes renal failure, and children who consume Pb content >16 g/dl suffer from profound IQ deficiency level 3.0 (13, 68, 69). The highest Cd content, 6.76 μg/g, was recorded in liver tissues and was higher than 3.50 μg/g in the liver of Nile tilapia caught from Ogun River, Southwestern Nigeria (1). The highest Pb content, 5.39 μg/g, was recorded in gills and was significantly lower than 30.83 μg/g in gills of Nile tilapia caught from Ogun River, Southwestern Nigeria (1). The possible source of Pb pollution could be fuel effluents from cruise ships (70). In agreement with our observations, non-essential trace elements Pb and Cd were slightly above the permissible limits according to FAO/WHO.

Micronuclei are synthesized during cell division (anaphase) when an entire chromosome or its part fails to become part of any daughter cell nucleus due to genetic damage (14, 71). MN test is a common technique to investigate implications caused by heavy metal contents on genotoxicity in erythrocytes (72). The key points to consider before starting to measure MN are (a) its diameter must be equal to or less than one-third of the central nucleus, (b) exist independently, (c) distinguished nuclear boundaries, and (d) stained the same as the central nucleus. Biosynthesis of MN in erythrocytes harvested from Nile tilapia was observed at ×1,000 magnification. We observed that the concentrations of MN induction in fishes were as follows: Mariout > Abbassa > Aswan, with frequencies of 5.24, 2.89, and 0.14% compared with baseline 0.65% (73). Higher MN number represents the highest level of trace elements contamination (74) and other trace pollutants in the body of aquatic animals (Figures 2A,B) (75). The micronuclei frequency increased progressively with the increase in the concentration of Zn, Cu, Pb, and Cd in Nile tilapia (14). Our results of the MN test about genotoxicity, water quality, the health status of aquatic animals, and their potential health risks to humans were consistent with previously published data (14, 72, 76). Similar findings have been reported in previous studies, such as the frequency of MN biosynthesis being highest in European minnow (*Phoxinus phoxinus*), eel (*Anguilla anguilla*) (77), Nile tilapia (78), crucian carp (79), and brown trout (*Salmo trutta*) (80).

Heavy metals stimulate overexpression of *Hsp70* in fish muscles but also causes a decline in antioxidants (81, 82). Heat shock protein 70 plays a key role in protein homeostasis and cellular stress response (15, 83). Hoq and Das (84) used *Hsp70* of fish as a biomarker of water quality in Bangladesh. Mohanty et al. (85) demonstrated that it is a sensitive indicator of exposure of aquatic animals to different pollutants and causes an abnormal increase of proteins in the nucleus and cytosol (86, 87). Due to trace elements contamination, Hsp70 protein bands in muscle tissues of Nile tilapia caught from Mariout and Abbassa was obvious but absent in muscle tissues of Nile tilapia caught from Aswan (Figure 3A), similar to AbdEl-Rahim et al. (88) and Deen (89). The expression of *Hsp70* was upregulated in the liver (Figures 3A,B) because it is responsible for the accumulation and detoxification of toxins (90) and performs multiple key functions as energy metabolism. Our results revealed that heavy metal contents in selected water resources were in the order Mariout > Abbassa > Aswan, and our findings are in accordance with Rasid et al. (91). Consequently, the consumption of Nile tilapia caught from Abbassa and Mariout Lake with higher Pb and Cd contents may have serious health implications for children, youth, and adults, excluding adults who consume Nile tilapia caught from Mariout. The Egyptian government had implemented National Water Resources Strategy (2017–2037) to curb the toxicity of fresh water resources which will approximately cost EGP 900 billion.

## Conclusions

The average trace elements in Nile tilapia caught from Mariout Lake, Abbassa, and Aswan of Egypt were significantly higher in the liver. The EDI of Pb, Cd, Cu, Mn, and Zn by eating Nile tilapia was less than the PTDI recommended by FAO/WHO. The concentrations of essential elements Cu, Zn, and Mn in Nile tilapia were lower, while toxic elements Pb and Cd in Nile tilapia caught from Abbassa and Mariout were slightly higher than the intake limit recommended by FAO, WHO, and EC. Micronuclei concentration in erythrocytes of Nile tilapia caught from different regions were Mariout > Abbassa > Aswan, and in different organs were liver > gills > muscle, which displayed genotoxic effect of trace elements. SDS-PAGE and qPCR analysis revealed upregulated expression of heavy metals responsive Hsp70 protein in muscle tissues of Nile tilapia caught from Mariout and Abbassa and low in Nile tilapia caught from Aswan. Thus, Nile tilapia, with other genotoxicity assessments, is a good indicator for aquatic environmental pollution due to its high sensitivity to low concentration of contaminants and hence is considered a bio-accumulator.

## Data Availability Statement

The original contributions presented in the study are included in the article/supplementary material, further inquiries can be directed to the corresponding authors.

## Ethics Statement

The animal study was reviewed and approved by National Institute of Oceanography and Fisheries, (NIOF), Egypt and American Psychological Association for Animal Research and Ethics in 2010-1.

## Author Contributions

AE-S, MA, JL, KY, and MS: conceptualization. AE-S and HA-K: experimental design. AE-S, HA-K, HE-S, and MS: data analysis and drawing figures. AE-S: writing—original draft. AE-S, HA-K, SS, HE-S, VY, HS, PM, XZ, JL, KY, MAAE, AMA, and MA: editing and proofreading. AE-S and MA: writing the final manuscript. All authors reviewed and approved the final submission.

## Funding

This study received funding from the High-level Talent Project of Yibin University (Grant No. 2018RC07). The funder was not involved in the study design, collection, analysis, interpretation of data, the writing of this article, or the decision to submit it for publication.

## Conflict of Interest

The authors declare that the research was conducted in the absence of any commercial or financial relationships that could be construed as a potential conflict of interest.

## Publisher's Note

All claims expressed in this article are solely those of the authors and do not necessarily represent those of their affiliated organizations, or those of the publisher, the editors and the reviewers. Any product that may be evaluated in this article, or claim that may be made by its manufacturer, is not guaranteed or endorsed by the publisher.

## References

[1] Ayanda IO Ekhator UI Bello OA. Determination of selected heavy metal and analysis of proximate composition in some fish species from Ogun River, Southwestern Nigeria. Heliyon. (2019) 5:e02512. 10.1016/j.heliyon.2019.e02512 31667377 PMC6812461

[2] Mas-Coma S Bargues M Valero M. Human fascioliasis infection sources, their diversity, incidence factors, analytical methods and prevention measures. Parasitology. (2018) 145:1665–99. 10.1017/S0031182018000914 29991363

[3] El-Sappah A Shawky A Sayed-Ahmad M Youssef M. Nile tilapia as bio indicator to estimate the contamination of water using SDS-PAGE and RAPDPCR techniques. Egypt J Genet Cytol. (2012) 41:209–27. 10.21608/ejgc.2012.10536

[4] El-Sheekh MM. Impact of water quality on ecosystems of the Nile River. In: The Nile River. Springer. (2016). p. 357–85. 10.1007/698_2016_97

[5] Authman MM Abbas HH Abbas WT. Assessment of metal status in drainage canal water and their bioaccumulation in Oreochromis niloticus fish in relation to human health. Environ Monit Assess. (2013) 185:891–907. 10.1007/s10661-012-2599-8 22451326

[6] El-Sappah AH Elrys AS Desoky El-S Zhao X Bingwen W El-Sappah HH. Comprehensive genome wide identification and expression analysis of MTP gene family in tomato (*Solanum lycopersicum*) under multiple heavy metal stress. Saudi J Biol Sci. (2021) 28:6946–56. 10.1016/j.sjbs.2021.07.073 34866994 PMC8626246

[7] Dórea JG. Persistent, bioaccumulative and toxic substances in fish: human health considerations. Sci Total Environ. (2008) 400:93–114. 10.1016/j.scitotenv.2008.06.017 18653214

[8] Badr AM Mahana NA Eissa A. Assessment of heavy metal levels in water and their toxicity in some tissues of Nile tilapia (Oreochromisniloticus) in River Nile basin at greater Cairo, Egypt. Global Veterinaria. (2014) 13:432–43. 10.5829/idosi.gv.2014.13.04.8561

[9] Varol M Sünbül MR. Environmental contaminants in fish species from a large dam reservoir and their potential risks to human health. Ecotoxicol Environ Safety. (2019) 169:507–15. 10.1016/j.ecoenv.2018.11.060 30472475

[10] El-Sappah AH Elbaiomy RG Elrys AS Wang Y Zhu Y Huang Q. Genome-wide identificationand expression analysis of metaltolerance protein gene familyinMedicago truncatulaundera broad range of heavy metalstress. Front Genet. (2021) 12:713224. 10.3389/fgene.2021.713224 34603378 PMC8482800

[11] Neeratanaphan L Kamollerd C Suwannathada P Suwannathada P Tengjaroenkul B. Genotoxicity and oxidative stress in experimental hybrid Catfish exposed to heavy metals in a Municipal Landfill Reservoir. Int J Environ Res Public Health. (2020) 17:1980. 10.3390/ijerph17061980 32192208 PMC7143293

[12] Andjelkovic M Buha Djordjevic A Antonijevic E Antonijevic B Stanic M Kotur-Stevuljevic J. Toxic effect of acute cadmium and lead exposure in rat blood, liver, and kidney. Int J Environ Res Public Health. (2019) 16:274. 10.3390/ijerph16020274 30669347 PMC6351928

[13] Seif MM Madboli A-N Marrez DA Aboulthana WM. Hepato-renal protective effects of Egyptian purslane extract against experimental cadmium toxicity in rats with special emphasis on the functional and histopathological changes. Toxicology Reports. (2019) 6:625–31. 10.1016/j.toxrep.2019.06.013 31367527 PMC6650623

[14] El-Sappah AHAM. Utilization of Micronucleus Test and Genetic Markers for Determining the Pollution of Nile tilapia (Oreochromis niloticus). Zagazig: Zagazig University (2013).

[15] El-Sappah AH Shawky A Sayed-Ahmad MS Youssef M. Estimation of heat shock protein 70 (Hsp70) gene expression in Nile tilapia (*Oreochromis niloticus*) using quantitative real-time PCR. Zagazig J Agric Res. (2017) 44:1003–15. 10.21608/zjar.2017.52300

[16] Abdel-Kader HH Mourad MH. Trace elements exposure influences proximate body composition and antioxidant enzyme activities of the species tilapia and catfish in Burullus Lake—Egypt: human risk assessment for the consumers. Environ Sci Pollut Res. (2020) 27:43670–81. 10.1007/s11356-020-10207-2 32740845

[17] Koca S Koca YB Yildiz S Gürcü B. Genotoxic and histopathological effects of water pollution on two fish species, Barbus capito pectoralis and Chondrostoma nasus in the Büyük Menderes River, Turkey. Biol Trace Element Res. (2008) 122:276–91. 10.1007/s12011-007-8078-3 18214389

[18] Jiang D Hu Z Liu F Zhang R Duo B Fu J. Heavy metals levels in fish from aquaculture farms and risk assessment in Lhasa, Tibetan Autonomous Region of China. Ecotoxicology. (2014) 23:577–83. 10.1007/s10646-014-1229-3 24671559

[19] Karadede-Akin H Ünlü E. Heavy metal concentrations in water, sediment, fish and some benthic organisms from Tigris River, Turkey. Environ Monit Assess. (2007) 131:323–37. 10.1007/s10661-006-9478-0 17171266

[20] Kris-Etherton PM Harris WS Appel LJ. Fish consumption, fish oil, omega-3 fatty acids, and cardiovascular disease. Circulation. (2002) 106:2747–57. 10.1161/01.CIR.0000038493.65177.94 12438303

[21] Marcon E Puech F. Measures of the geographic concentration of industries: improving distance-based methods. J Econ Geogr. (2010) 10:745–62. 10.1093/jeg/lbp056

[22] Glusczak L Dos Santos Miron D Crestani M Braga Da Fonseca M Araújo Pedron FD Duarte MF. Effect of glyphosate herbicide on acetylcholinesterase activity and metabolic and hematological parameters in piava (*Leporinus obtusidens*). Ecotoxicol. Environ. Saf. (2006) 65:237–41. 10.1016/j.ecoenv.2005.07.017 16174533

[23] Modesto KA Martinez CB. Roundup® causes oxidative stress in liver and inhibits acetylcholinesterase in muscle and brain of the fish *Prochilodus lineatus*. Chemosphere. (2010) 78:294–9. 10.1016/j.chemosphere.2009.10.047 19910015

[24] Abdel-Kader HH Mourad MH. Bioaccumulation of heavy metals and physiological/histological changes in gonads of Catfish (Clarias gariepinus) inhabiting lake Maryout, Alexandria, Egypt. Egyptian J Aquatic Biol Fish. (2019) 23:363–77. 10.21608/ejabf.2019.32036

[25] Francisco CDM Bertolino SM De Oliveira Junior RJ Morelli S Pereira BB. Genotoxicity assessment of polluted urban streams using a native fish Astyanax altiparanae. J Toxicol Environ Health A. (2019) 82:514–23. 10.1080/15287394.2019.1624235 31140379

[26] Baršiene J Butrimavičiene L Grygiel W Lang T Michailovas A Jackunas T. Environmental genotoxicity and cytotoxicity in flounder (*Platichthys flesus*), herring (*Clupea harengus*) and Atlantic cod (*Gadus morhua*) from chemical munitions dumping zones in the southern Baltic Sea. Marine Environ Res. (2014) 96:56–67. 10.1016/j.marenvres.2013.08.012 24064039

[27] Heddle J Cimino M Hayashi M Romagna F Shelby M Tucker J. Micronuclei as an index of cytogenetic damage: past, present, and future. Environ Mol Mutagen. (1991) 18:277–91. 10.1002/em.2850180414 1748091

[28] Çavaş T Ergene-Gözükara S. Micronucleus test in fish cells: a bioassay for *in situ* monitoring of genotoxic pollution in the marine environment. Environ Mol Mutagen. (2005) 46:64–70. 10.1002/em.20130 15880416

[29] Labrada-Martagón V Teneriá FAM Zenteno-Savín T. Standardized micronucleus assay for peripheral blood from sea turtles. Chelonian Conserv Biol. (2019) 18:175–86. 10.2744/CCB-1373.1

[30] Bustin SA Benes V Garson JA Hellemans J Huggett J Kubista M. The MIQE guidelines: Minimum information for publication of quantitative real-time PCR experiments. Clin Chem. (2009) 55:611–22. 10.1373/clinchem.2008.112797 19246619

[31] Hamdy A. Protein analysis and heavy metals accumulation in muscles of wild and farmed Nile tilapia (Oreochromis niloticus). Assiut Univ Bull Environ Res. (2017) 20:11–22. 10.21608/auber.2017.145812

[32] AVMA (American Veterinary Medical Association). (2020). Guidelines on Euthanasia (formerly the Report of the AVMA Panel on Euthanasia). Available online at: https://www.avma.org/sites/default/files/2020-01/2020-Euthanasia-Final-1-17-20.pdf (accessed April 21, 2020).

[33] Neiffer DL Stamper MA. Fish sedation, anesthesia, analgesia, and Euthanasia: considerations, methods, and types of drugs. ILAR J. (2009) 50:343–60. 10.1093/ilar.50.4.343 19949251

[34] Medeiros RJ dos Santos LMG Freire AS Santelli RE Braga AMC Krauss TM. Determination of inorganic trace elements in edible marine fish from Rio de Janeiro State, Brazil. Food Control. (2012) 23:535–41. 10.1016/j.foodcont.2011.08.027

[35] EU. Commission Regulation as regards heavy metals, Directive, 2001/22/EC, No: 466. FAO/WHO, 2004. Summary of Evaluations Performed by the Joint FAO/WHO Expert Committee on Food Additives (JECFA 1956–2003), (First Through Sixty First Meetings). ILSI Press International Life Sciences Institute (2001). Available online at: https://ec.europa.eu/

[36] WHOF. Vitamin and Mineral Requirements in Human Nutrition. Geneva: World Health Organization and Food and Agriculture Organization of the United Nations (2004).

[37] GAFRD. General Authority for Fish Resources Development, Yearbook of Fishery Statistics Cairo-Egypt. GAFRD (2015).Available online at:https://www.gafrd.org/posts/

[38] Hooftman RN De Raat W. Induction of nuclear anomalies (micronuclei) in the peripheral blood erythrocytes of the eastern mudminnow *Umbra pygmaea* by ethyl methanesulphonate. Mutation Research Letters. (1982) 104:147–52. 10.1016/0165-7992(82)90136-1 7078568

[39] Fenech M Chang WP Kirsch-Volders M Holland N Bonassi S Zeiger E. HUMN project: detailed description of the scoring criteria for the cytokinesis-block micronucleus assay using isolated human lymphocyte cultures. Mutation Res/Genetic Toxicol Environ Mutagenesis. (2003) 534:65–75. 10.1016/S1383-5718(02)00249-8 12504755

[40] El-Deeb AM El-Sappah AH Arisha MH. Efficiency of some bionematicides against root-knot nematode Meloidogyne incognita on three tomato cultivars under greenhouse conditions. Zagazig J Agric Res. (2018) 45:2. 10.21608/zjar.2018.47739

[41] Wang J Abbas M Wen Y Niu D Wang L Sun Y. Selection and validation of reference genes for quantitative gene expression analyses in black locust (*Robinia pseudoacacia* L.) using real-time quantitative PCR. PLoS ONE. (2018) 13:e0193076. 10.1371/journal.pone,.0193076 29529054 PMC5846725

[42] Abbas M Peszlen I Shi R Kim H Katahira R Kafle K. Involvement of CesA4, CesA7-A/B and CesA8-A/B in secondary wall formation in Populus trichocarpa wood. Tree Physiol. (2020) 40:73–89. 10.1093/treephys/tpz020 31211386

[43] Shi Z Zhang H Liu Y Xu M Dai J. Alterations in gene expression and testosterone synthesis in the testes of male rats exposed to perfluorododecanoic acid. Toxicol Sci. (2007) 98:206–15. 10.1093/toxsci/kfm070 17400581

[44] Livak KJ Schmittgen TD. Analysis of relative gene expression data using real-time quantitative PCR and the 2–ΔΔCT method. Methods. (2001) 25:402–8. 10.1006/meth.2001.1262 11846609

[45] Cheng Y Wang L Abbas M Huang X Wang Q Wu A. MicroRNA319-mediated gene regulatory network impacts leaf development and morphogenesis in poplar. Forestry Res. (2021) 1:1–10. 10.48130/FR-2021-0004 PMC11524277 39524520

[46] Gheorghe S Stoica C Vasile GG Nita-Lazar M Stanescu E Lucaciu IE. Metals toxic effects in aquatic ecosystems: modulators of water quality. Water Qual. (2017) 60–89. 10.5772/65744

[47] Shreadah MA El-Rayis OA Shaaban NA. et al. Water quality assessment and phosphorus budget of a lake (Mariut, Egypt) after diversion of wastewaters effluents. Environ Sci Pollut Res. (2020) 27:26786–99. 10.1007/s11356-020-08878-y 32382910

[48] Abu El-Magd SA Taha TH Pienaar HH Breil P Amer RA Namour P. Assessing heavy metal pollution hazard in sediments of Lake Mariout, Egypt. J Afr Earth Sci. (2021) 176:104116. 10.1016/j.jafrearsci.2021.104116

[49] Boyd CE Tucker CS. Ecology of aquaculture ponds. In: Pond Aquaculture Water Quality Management. Berlin: Springer. (1998). p. 8–86.

[50] Soltan M Awadallah R. Chemical survey on the River Nile water from Aswan into the outlet. J Environ Sci Health A. (1995) 30:1647–58. 10.1080/10934529509376293

[51] Al-Fartusie FS Mohssan SN. Essential trace elements and their vital roles in human body. Indian J Adv Chem Sci. (2017) 5:127–36. 10.22607/IJACS.2017.50300315285149

[52] Chi QQ Zhu GW Langdon A. Bioaccumulation of heavy metals in fishes from Taihu Lake, China. J Environ Sci. (2007) 19:1500–4. 10.1016/S1001-0742(07)60244-7 18277656

[53] Baharom ZS Ishak MY. Determination of heavy metal accumulation in fish species in Galas River, Kelantan and Beranang mining pool, Selangor. Proc Environ Sci. (2015) 30:320–5. 10.1016/j.proenv.2015.10.057

[54] Kiral G. Mutlulugun göstergeleri ve hanehalki için bazi sonuçlar: adana örnegi merve maşa. EURO ASIA. (2019). p. 618.

[55] El-Moselhy KM Othman AI El-Azema HA El-Metwally MEA. Bioaccumulation of heavy metals in some tissues of fish in the Red Sea, Egypt. Egypt J Basic Appl Sci. (2017) 1:97–105. 10.1016/j.ejbas.2014.06.00133732073

[56] Avila DS Puntel RL Aschner M. Manganese in health and disease. In: Interrelations Between Essential Metal Ions and Human Diseases. Berlin: Springer(2013). p. 199–227. 10.1007/978-94-007-7500-8_7 PMC6589086 24470093

[57] Mezzaroba L Alfieri DF Simão ANC Reiche EMV. The role of zinc, copper, manganese and iron in neurodegenerative diseases. Neurotoxicology. (2019) 74:230–41. 10.1016/j.neuro.2019.07.007 31377220

[58] Adeyeye EI Akinyugha NJ Fesobi ME Tenabe VO. Determination of some metals in Clarias gariepinus (Cuvier and Vallenciennes). *Cyprinus carpio* (L) and *Oreochromis niloticus* (L) fishes in a polyculture fresh water pond and their environments *Aquaculture*. (1996) 147:205–14. 10.1016/S0044-8486(96)01376-2

[59] Sivaperumal P Sankar T Nair PV. Heavy metal concentrations in fish, shellfish and fish products from internal markets of India vis-a-vis international standards. Food Chem. (2007) 102:612–20. 10.1016/j.foodchem.2006.05.041

[60] Prasad AS. Discovery of human zinc deficiency: Its impact on human health and disease. Adv Nutr. (2013) 4:176–90. 10.3945/an.112.003210 23493534 PMC3649098

[61] Hill GM Shannon MC. Copper and zinc nutritional issues for agricultural animal production. Biol Trace Elem Res. (2019) 188:148–59. 10.1007/s12011-018-1578-5 30612303 PMC6373331

[62] Bartzatt R. Neurological impact of zinc excess and deficiency *in vivo*. Eur J Nutr Food Safety. (2017) 155–60. 10.9734/EJNFS/2017/35783

[63] Aydemir TB Blanchard RK Cousins RJ. Zinc supplementation of young men alters metallothionein, zinc transporter, and cytokine gene expression in leukocyte populations. Proc Nat Acad Sci. (2006) 103:1699–704. 10.1073/pnas.0510407103 16434472 PMC1413653

[64] FDAF. Guidance for Industry: Bioanalytical Method Validation. (2001). Available online at: http://www.fda.gov/cder/Guidance/4252fnl.pdf

[65] Atrián-Blasco E Conte-Daban A Hureau C. Mutual interference of Cu and Zn ions in Alzheimer's disease: perspectives at the molecular level. Dalton Trans. (2017) 46:12750–9. 10.1039/C7DT01344B 28937157 PMC5656098

[66] Taweel A Shuhaimi-Othman M Ahmad A. Assessment of heavy metals in tilapia fish (*Oreochromis niloticus*) from the Langat River and Engineering Lake in Bangi, Malaysia, and evaluation of the health risk from tilapia consumption. Ecotoxicol Environ Safety. (2013) 93:45–51. 10.1016/j.ecoenv.2013.03.031 23642778

[67] Skidmore J. Toxicity of zinc compounds to aquatic animals, with special reference to fish. Quart Rev Biol. (1964) 39:227–48. 10.1086/404229 14206619

[68] Järup L Alfvén T. Low level cadmium exposure, renal and bone effects–the OSCAR study. Biometals. (2004) 17:505–9. 10.1023/b:biom.0000045729.68774.a1 15688854

[69] Kumar A Kumar A M M S CP Chaturvedi AK Shabnam AA Subrahmanyam G. Lead Toxicity: Health hazards, influence on food chain, and sustainable remediation approaches. International Journal of Environmental Research and Public Health. (2020) 17:2179. 10.3390/ijerph17072179 32218253 PMC7177270

[70] Ahamed M Siddiqui MKJ. Environmental lead toxicity and nutritional factors. Clin Nutr. (2007) 26:400–8. 10.1016/j.clnu.2007.03.010 17499891

[71] Luzhna L Kathiria P Kovalchuk O. Micronuclei in genotoxicity assessment: From genetics to epigenetics and beyond. Frontiers in Genetics. 4:131. 10.3389/fgene.2013.00131 23874352 PMC3708156

[72] Al-Sabti K Metcalfe CD. Fish micronuclei for assessing genotoxicity in water. Mutation Res/Genet Toxicol. (1995) 343:121–35. 10.1016/0165-1218(95)90078-0 7791806

[73] Da Silva Souza T Fontanetti CS. Micronucleus test and observation of nuclear alterations in erythrocytes of Nile tilapia exposed to waters affected by refinery effluent. Mut Res/Genet Toxicol Environ Mutagenesis. (2006) 605:87–93. 10.1016/j.mrgentox.2006.02.010 16678473

[74] Kašuba V Milić M ŽeljeŽić D Mladinić M Pizent A Kljaković-Gašpić Z. Biomonitoring findings for occupational lead exposure in battery and ceramic tile workers using biochemical markers, alkaline comet assay, and micronucleus test coupled with fluorescence *in situ* hybridization. Arh Hig Rada Toksikol. (2020) 71:339–52. 10.2478/aiht-2020-71-3427 33410779 PMC7968510

[75] Dourado PLR Rocha MPD Roveda LM Raposo Junior JL Cândido LS Cardoso CAL. Genotoxic and mutagenic effects of polluted surface water in the midwestern region of Brazil using animal and plant bioassays. Genet Mol Biol. (2017) 40:123–33. 10.1590/1678-4685-gmb-2015-0223 27801481 PMC5409763

[76] Ali D Almarzoug MH Al-Ali H Samdani M Hussain S Alarifi S. Fish as bio indicators to determine the effects of pollution in river by using the micronucleus and alkaline single cell gel electrophoresis assay. J King Saud Univ Sci. (2020) 32:2880–5. 10.1016/j.jksus.2020.07.012

[77] Sanchez-Galan S Linde A Ayllon F Garcia-Vazquez E. Induction of micronuclei in eel (*Anguilla anguilla* L.) by heavy metals. Ecotoxicol Environ Safety. (2001) 49:139–143. 10.1006/eesa.2001.2048 11386727

[78] Chandra P Khuda-Bukhsh A. Genotoxic effects of cadmium chloride and azadirachtin treated singly and in combination in fish. Ecotoxicol Environ Safety. (2004) 58:194–201. 10.1016/j.ecoenv.2004.01.010 15157573

[79] Arkhipchuk V Garanko N. Using the nucleolar biomarker and the micronucleus test on *in-vivo* fish fin cells. Ecotoxicol Environ Saf. (2005) 62:42–52. 10.1016/j.ecoenv.2005.01.001 15978290

[80] Sanchez-Galan S Linde A Garcia-Vazquez E. Brown trout and European minnow as target species for genotoxicity tests: differential sensitivity to heavy metals. Ecotoxicol Environ Saf. (1999) 43:301–4. 10.1006/eesa.1999.1794 10381308

[81] Padmini E Rani MU. Thioredoxin and HSP90α modulate ASK1–JNK1/2 signaling in stressed hepatocytes of Mugil cephalus. Comparat Biochem Physiol C Toxicol Pharmacol. (2010) 151:187–93. 10.1016/j.cbpc.2009.10.004 19861173

[82] Authman MM Zaki MS Khallaf EA Abbas HH. Use of fish as bio-indicator of the effects of heavy metals pollution. J Aquacult Res Dev. (2015) 6:1–13. 10.4172/2155-9546.1000328

[83] Keller JM Escara-Wilke JF Keller ET. Heat stress-induced heat shock protein 70 expression is dependent on ERK activation in zebrafish (*Danio rerio*) cells. Comparat Biochem Physiol A Mol Integr Physiol. (2008) 150:307–14. 10.1016/j.cbpa.2008.03.021 18467140 PMC2507762

[84] Hoq T Das AR. Fish stress protein: an approach for biomonitoring of water quality in Bangladesh. Am J Zool Res. (2017) 5:24–8.

[85] Mohanty BP Mahanty A Mitra T Parija SC Mohanty S. Heat shock proteins in stress in teleosts. In: Regulation of Heat Shock Protein Responses. Berlin: Springer. (2018). p. 71–94.

[86] Sherman MY Goldberg AL. Cellular defenses against unfolded proteins: a cell biologist thinks about neurodegenerative diseases. Neuron. (2001) 29:15–32. 10.1016/S0896-6273(01)00177-5 11182078

[87] Padmini E. Physiological adaptations of stressed fish to polluted environments: role of heat shock proteins. Rev Environ Contam Toxicol. (2010) 206:1–27. 10.1007/978-1-4419-6260-7_1 20652666

[88] AbdEl-Rahim WM Khalil WK Eshak MG. Evaluation of the gene expression changes in Nile tilapia (*Oreochromis niloticus*) as affected by the bio-removal of toxic textile dyes from aqueous solution in small-scale bioreactor. Environmentalist. (2010) 30:242–53. 10.1007/s10669-010-9268-7

[89] Deen SE. Accumulation of a 70 kDa stress protein in the Nile tilapia, Oreochromis niloticus, and its use as a biomarker of Cu exposure. Egyptian J Aquatic Biol Fish. (2006) 10:19–31. 10.21608/ejabf.2006.1846

[90] Rajeshkumar S Mini J Munuswamy N. Effects of heavy metals on antioxidants and expression of HSP70 in different tissues of Milk fish (*Chanos chanos*) of Kaattuppalli Island, Chennai, India. Ecotoxicol Environ Saf. (2013) 98:8–18. 10.1016/j.ecoenv.2013.07.029 24021871

[91] Rasid MHFA Deris ZM Shazili NAM De Boeck G Wong LL. Putative roles for metallothionein and HSP70 genes in relation with heavy metal accumulation and parasitic cymothoid in the fish Nemipterus furcosus. Arch Environ Contamin Toxicol. (2016) 71:530–40. 10.1007/s00244-016-0310-8 27638714

